# Framing the social in biomedical HIV prevention trials: a 20-year retrospective

**DOI:** 10.1186/1758-2652-14-S2-S3

**Published:** 2011-09-27

**Authors:** Kathleen M MacQueen

**Affiliations:** 1 Behavioral and Social Sciences, FHI360, 2224 ENC Hwy 54, Durham, NC 27713 JSA

## Abstract

Biomedical research is critical to identifying effective and safe interventions, such as vaccines, microbicides, male circumcision and antiretrovirals, for prevention. Funding for clinical prevention trials is highly competitive and the benchmarks of success ultimately reduce to quickly enrolling a select group of people at risk, keeping them enrolled, and inducing them to be compliant with trial requirements - all at the lowest cost possible. Juxtaposed with this reality is the fact that HIV is situated with poverty, exploitation, assaults on human dignity, and human rights abuses. The result is a complex web of ethical challenges that are socially constructed along lines of wealth and power. While social science research methods are commonly employed to examine such topics, they have played a marginal role in biomedical HIV prevention research. Why? To answer this question, a core set of persistent interlocking social, behavioural and ethical challenges to biomedical HIV prevention research are described. A critique is offered on how the social has been framed relative to the behavioural, ethical and biomedical components. Examples of how this framing has devalued social knowledge are provided, including the conflation of qualitative research with anecdotal reporting, a bias toward brevity and accuracy over external validity, and difficulties in distinguishing between a moral understanding of social norms and achieving a moral outcome when confronted with ethical challenges in research. Lastly, opportunities are identified for enhancing the success of biomedical HIV prevention research through development of a coherent programme of social science research. Recommendations are offered for reframing the social as a valid domain of scientific inquiry in this highly applied and interdisciplinary context.

## Introduction

Biomedical research is critical to identifying effective and safe interventions, such as vaccines, microbicides, male circumcision and antiretrovirals, for HIV prevention. It is also a resource-intensive endeavour in terms of funding, clinical infrastructure, oversight and scientific capacity. The nature of the HIV epidemic is such that prevention research is situated with poverty, exploitation, assaults on human dignity, and human rights abuses. The result is a complex web of research and intervention challenges that are socially constructed along lines of wealth and power. A wide range of disciplines, collectively known as the social sciences, have long made such phenomena an object of study and discourse: anthropology, sociology, political science, history, economics and geography, to name a few. Yet despite the fact that social science research methods are commonly employed to examine such topics, they have played a marginal role in biomedical HIV prevention research.

What follows is a retrospective based on my personal experiences as a social scientist actively collaborating with biomedical HIV prevention researchers conducting vaccine, microbicide and pre-exposure prophylaxis (PrEP) trials. Beginning in the early 1990s, I was one of many US government scientists tasked with planning for the first HIV vaccine trials. Those leading the effort recognized the need to address a wide range of social and behavioural questions [1]. Would AIDS stigma deter people from participating? Could we effectively recruit and screen trial participants when this required asking them about stigmatized sexual and drug-using behaviours? Could we successfully enrol and retain people who were at high risk for HIV, but who were also marginalized in their own communities? Could we prevent people in a vaccine trial from believing that they were protected and taking risks they otherwise would not? How could biomedical HIV prevention researchers overcome the distrust of science and government-funded research that grew out of an unfortunate and recurring history of medical exploitation of the vulnerable?

When the first HIV vaccine preparatory studies were fielded in various US cities in 1993, the lead investigators reflected the multidisciplinary nature of the questions to be addressed: epidemiology, medicine, psychology, sociology, anthropology, public health and social work. This was despite the fact that overall leadership for the work was housed in the solidly biomedical National Institute of Allergy and Infectious Diseases at the National Institutes of Health (NIH).

The approach was, in retrospect, a groundbreaking endeavour. To be sure, the ground was, and remains, rocky and tangled with deeply established roots. In my experience, the social sciences have had the greatest difficulty establishing themselves in this terrain. Yet the social dimensions are among the most critical for successful prevention trial implementation and translation of trial findings to effective prevention programmes. Through this retrospective, I hope to throw some light on the obstacles, the successes and the opportunities for social scientists in this challenging field.

### Biomedical HIV prevention: a brief overview

Because HIV is a global epidemic, efforts similar to the early US preparatory studies emerged elsewhere. The World Health Organization (WHO) (and later UNAIDS) and NIH independently established international programmes to build capacity for HIV vaccine trials in Africa, Asia and Latin America. European nations, Australia and Canada similarly developed national and international research agendas. Scientific centres with strong in-country leadership emerged in Thailand, Brazil, South Africa and elsewhere [1-3]. The biomedical HIV prevention research agenda diversified to include micro-bicides, interventions to prevent mother to child transmission, medical male circumcision, treatment of sexually transmitted infections that facilitated HIV transmission, PrEP, and early antiretroviral treatment of HIV infection to reduce transmission by reducing viral load. Complex partnerships with pharmaceutical companies were developed, scientific non-profits formed with the goal of accelerating the development of vaccines and microbicides, and advocacy groups emerged to promote, support and build awareness for biomedical HIV prevention.

The large-scale phase III HIV vaccine effectiveness trials that were the impetus for the early US preparedness studies were not implemented. In a controversial and hotly debated decision-making process in 1995, NIH concluded that the results from smaller phase II trials did not support further testing. As Cohen describes in detail in a chapter titled “Perpetual Uncertainty” is his 2001 book on the search for an AIDS vaccine, the decision reflected concerns about community readiness, as well as scientific uncertainties [4].

As the years unfolded, it became clear that controversy would be the norm for biomedical HIV prevention. Since the mid-1990s, dozens of biomedical HIV prevention trials have been implemented, most with flat or negative results [5]. There have been highly charged debates about the plausibility, utility, ethics, viability and acceptability of every biomedical intervention tested in phase II and III trials [4,6]. Despite these challenges, by mid-2011, we had evidence that oral PrEP and antiretroviral-based vaginal gels were partially protective for at least some populations when used consistently, that a two-vaccine combination may be marginally protective, that medical circumcision reduced infection rates in men by 60%, that early antiretroviral treatment could reduce transmission by reducing viral load, and that the proper use of antiretrovirals could virtually eliminate mother to child HV transmission in the absence of breastfeeding [7-14]. Despite all the controversies, flat results and failures, biomedical options clearly are an important and growing part of the HIV prevention toolkit.

## Discussion

### Interlocking challenges

Most biomedical HIV prevention research is funded publicly or through non-profit foundations, yet the funding context is highly competitive and the benchmarks of success ultimately reduce to the same three that drive profit-driven research: quickly enrolling a select group of people at risk, keeping them enrolled, and inducing them to be compliant with trial requirements - all at the lowest cost possible. As noted previously, this reality is situated within a complex web of persistent interlocking social, behavioural and ethical challenges.

To a large extent, stigma sits at the core of the interlock. In his seminal sociological work, Goffman identified three categories of stigma: physiological, including disease; behavioural; and social [15]. HIV sits at the intersection of all three: it is a deadly and debilitating disease; it is transmitted via behaviours subject to moral judgment, including sex and drug use; and in non-generalized epidemics, it is often most prevalent among groups subject to multiple forms of discrimination and marginalization. Stigma serves to perpetuate the HIV epidemic by creating barriers to prevention and treatment and compounding the struggles of individuals, families and communities affected [16,17].

Distrust of medical research, government agencies and international entities is another core component of the interlock. The uncontested examples of exploitation and harm are too numerous to cite here, ranging from Nazi experimentation on concentration camp prisoners in the World War II era to the Tuskegee syphilis study among impoverished African Americans in the post-war era to the post-2000 Discovery Laboratories’ proposal to conduct a placebo-controlled trial of an experimental treatment for Respiratory Distress Syndrome in infants in Latin America despite the existence of approved treatment drugs in the US [18,19].

The scientists involved in these and other exploitative studies typically cited contributions to a greater good as justification. In some cases, arguments were made that no real harm was done because the research subjects were lacking access to adequate care anyway, were given other benefits (e.g., coverage of burial costs in the Tuskegee study), or were going to die regardless of participation in research. More politely put, many of the researchers did not actively increase the level of harm experienced by participants, but neither did they use the opportunity to mitigate harm with the resources and knowledge at hand via the research.

Most of the major controversies surrounding HIV prevention trials reference this history because the trials are situated on the jagged divide between those with wealth and power and those without [20,21]. Perhaps unique to the HIV context, the controversies ultimately brought researchers and advocates together to find ways to bridge the divide. The result has been open dialogue about the most challenging ethical dilemmas, stronger partnerships between civil society and research, and innovative solutions to improving healthcare access for participants in HIV prevention trials [22,23].

### Framing the social

Ultimately the persistent interlocking social, behavioural and ethical challenges are about social relationships. Yet they have been framed primarily in behavioural and ethical terms. The result is an oversimplification of the social processes at work and reliance on a limited set of social science methods to explore and inform decision making. Examples of how this framing has devalued social knowledge include the conflation of qualitative research with anecdotal reporting, the privileging of brevity and accuracy over external validity, and difficulties in distinguishing between a moral understanding of social norms and achieving a moral outcome when confronted with ethical challenges in research.

Social scientists are now integrated as members of biomedical HIV prevention trial research teams, yet social science is minimally integrated with the science of biomedical HIV prevention. This is seen most notably in a trend toward funding biomedical researchers to lead increasingly complex intervention designs that include social interventions (with an emphasis on community, peer and household relationships). However, the trials rarely engage in in-depth, reflexive social science research on the broader implications of interventions for the communities and health systems within which they may be delivered.

### Conflation of qualitative research and anecdotal reports

There are two misconceptions about qualitative research that create barriers to the development of a systematic social science research agenda related to biomedical HIV prevention. First is a prevalent assumption that qualitative research is quick, cheap and simple to conduct. Unfortunately, social scientists can be their own worst enemies on this issue.

For example, some ethnographers describe their approach as “deep hanging out” following a popular article title by noted anthropologist Clifford Geertz [24]. Those trained in ethnographic research understand the nuances embedded in that phrase and realize that it is an attempt to explain a complex method by reference to a superficially similar social activity: it looks like loitering to the untrained eye, but is actually work. To people who spend long days in clinics and laboratories trying to figure out why one person is infected with HIV and another is not, the nuance of “deep hanging out” is lost. It sounds either like self-serving rationalization or something simple that a motivated clinician could do in her spare time. She can hang out in the waiting room or the local market, talking to people and hearing their stories. She can invite community members and study participants to a small group meeting, call it a focus group, and see what they think about the research being done out of the clinic.

Medical practitioners and researchers write and submit anecdotal reports to their journals all the time, and have developed standardized guidance and formats for presenting case examples [25]. Patient case reports are considered “valuable resources of new and unusual information that may lead to vital research” but are not considered to be research findings themselves [26]. The “unique and illustrative example” is well received and makes for interesting presentations.

These are not the same as doing a well-designed qualitative study that explicitly addresses issues of subjective bias and perspective. Biomedical researchers understand the potential for bias in anecdotal reports, but have developed a narrow approach for how to address it [26]. As social scientists, we need to do a better job of framing what we do relative to the standards of biomedicine if we want our findings to be given the weight they deserve.

The conflation of qualitative research with anecdotal reports and informal observation is also seen in confusion about the distinction between doing social science research on the one hand and, on the other, using the good participatory practices in biomedical research outlined in the guidance developed by AVAC and UNAIDS [23]. This is a fuzzy area because good participatory practices are often informed by social science research, and social science research is often conducted using participatory principles. There is thus a natural relationship between social science research and socially informed biomedical research. But there is also an important difference that is ignored to the detriment of both.

Presenting evidence of the how and why of stigma, documenting its impact on life choices and health incomes, and developing case examples of the way gender dynamics compound the effects of stigma on households are examples of research that can inform advocacy for research participants, guidelines for trial implementation and the roll out of comprehensive biomedical interventions. But it entails more than telling a powerful story about a person’s life. Social science research seeks to contextualize the story within competing social agendas, to relate it to other stories and to understand if it is typical or exceptional. Advocacy seeks to contextualize the story within an admittedly biased perspective and with a stated end goal in mind. Advocacy entails the selective use of information. There is an inherent problem with conflating this advocacy end goal with the means of social science research.

I am not arguing that social scientists should not engage in advocacy or that they should discourage the use of their findings for advocacy. HIV researchers in general are among some of the most effective and powerful advocates for the people and communities impacted by the disease and its drivers. But social science warrants a platform and recognition as science. A review of the scientific programme for almost any major HIV conference in the past 20 years is likely to show that social science contributions are either absent or rolled together with advocacy. The result is a social science standard limited to reportage and commentary, with few structured opportunities to generate a synthesis of cumulative findings that point with some clarity toward next steps. This is in stark contrast to the standard for clinical, laboratory and behavioural HIV research.

### Privileging of brevity and accuracy over external validity

When social science is not being lumped with advocacy, it is often redefined as a sub-discipline of behavioural science. Again, there is a natural relationship between the two and important work that straddles them, but they are not the same. Social science pursues knowledge about people in relationship to each other; behavioural science pursues understanding of people as individuals. When a behavioural scientist considers social context, it is to understand how it influences an individual’s behaviour. When a social scientist considers behaviour, it is to understand how it simultaneously emerges from and influences the dynamics of human relationships at multiple levels. Context is a predictor or a modifier for behavioural scientists, and a dynamic set of interlocking systems for social scientists. They are different paradigms.

A social science perspective on biomedical interventions brings to the fore important questions about how such interventions function as components of systems. Clinical HIV prevention trials are peculiar endeavours in that they attempt to maximize the generalizability of the biomedical findings concerning efficacy or effectiveness by controlling for the influence of environment and behaviour (including the behaviour of the research team, as well as that of the participant). Generalizability of the process by which the intervention is implemented has been dismissed as infeasible due to the fact that clinical trials are very different contexts from the real-world context within which successful biomedical interventions will need to be deployed. This dismissal misses the point of external validity, which includes the opportunity to assess the gradient of similarity between the clinical research and the programme implementation contexts to identify the degree of generalizability.

For example, many research teams have developed *ad hoc* procedures to improve microbicide adherence in the context of gender power dynamics in a range of cultural contexts. A social analysis across multiple trials could describe the potential generalizability of such procedures to the programme implementation context. Additionally, it could point toward development of combination prevention strategies that include structural components to address gender-based drivers of risk together with biomedical components to reduce biological vulnerabilities [27]. Latkin and colleagues offer a comprehensive framework for considering how to describe the interconnected and dynamic processes of change across multiple levels in dynamic social systems, which could provide a helpful starting place for this kind of analysis [28].

Lumping social science with behavioural science fosters a reduction of research questions to the level of the individual. Structural constraints within the clinical trials research context then further limits the scope of the research. Trial funding is generally tight and the research team is under pressure to begin enrolling participants as soon as possible. In an effort to minimize costs and implementation delays, data collection instruments are often limited in content, unsophisticated in design, and rarely piloted and validated prior to being implemented. Simultaneously, there is a presumption that the primary limiting factor for the utility of the data is the numeric accuracy of self-reported information. The result has been duplicative documentation of a narrow set of behavioural issues related to behavioural risk, acceptability, adherence, comprehension and getting study participants to answer questions truthfully and accurately. The data are used primarily to fix trial implementation problems on the fly, to demonstrate that problems were resolved, or to address political challenges for the clinical investigator.

These are important issues and warrant attention. But because they are primarily defined as practical clinical research questions, there are barriers to using the findings as a foundation for deepening our understanding of either the behavioural or social dimensions of biomedical prevention. Worse, the resultant findings are minimally informative for interpreting trial outcomes. The perverse outcome of this devaluing of standards is a questioning of the value of both social and behavioural science for biomedical prevention.

Despite the barriers, there are encouraging examples of a more nuanced approach, particularly in the field of microbicides with its strong history of community advocacy and linkages to women’s health and gender equity [29]. The MDP301 Pro2000 microbicide trial included a range of quantitative and qualitative data collection strategies with participants and their partners that supported extensive use of triangulation [30,31]. The Carraguard microbicide research programme included assessment of social issues in earlier stage trials with incorporation of findings into later trial design [32]. The recently closed FEM-PrEP oral PrEP trial included a comprehensive social and behavioural research component with community mapping, quantitative and qualitative data collection with participants and stakeholders, and use of social marketing methods to inform planning for potential roll out if the intervention proved successful [33-36].

### Distinguishing moral understanding and moral outcomes

Many of the social issues surrounding biomedical HIV prevention trials are framed as primarily ethical issues or challenges. This has resulted in a greater valuing of philosophical and logical argumentation over observation and evaluation for understanding and addressing the social dimensions of ethical challenges. Contextual understanding of the lives of people and the values they share -understanding why values differ from one context to another and the extent to which competing values may exist in a given setting - has at times been re-labelled as ethical relativism and condemned as supportive of exploitation, discrimination and human rights abuses. Or, it has been expropriated, for example, by facile arguments that interpret greater respect for local culture as “ethical” justification for requiring women to obtain their husbands’ consent to participate in research. Uninformed by a more nuanced social analysis, such arguments have promoted a disregard for women’s autonomy in some quarters.

In contrast, through analysis of the discourse around this issue by a wide range of stakeholders in diverse research communities, social science research on the underlying gender power dynamics has highlighted a space where respectful engagement can be negotiated without requiring complacency in the face of inequity and discrimination, and certainly without excusing the willful exploitation of inequities between well-resourced researchers and poorly resourced communities [27,37,38]. As Macklin notes, “It is one thing to provide an explanation of why an individual or entire culture holds certain beliefs and acts in certain ways. It is quite another thing to provide a justification for those beliefs and actions” [[6]; see page 24]. I would add that it is yet another thing to create a path from one set of established beliefs and norms about what is right to an alternative set in the limited context of biomedical research. Understanding local moral systems is an essential component to any effort seeking to achieve moral outcomes defined at a level of global discourse.

Beyond the context of HIV prevention clinical trials, social scientists have been significant contributors to the growing field of empirical and applied research ethics. For example, the journal, Social Science and Medicine, published a special issue on social science and bioethics in the African context [39]. Specific to HIV prevention trials, we can cite empirical research on informed consent, community engagement and ancillary care that has informed the conduct of clinical trials [38,40-43]. Nonetheless, there have been few social analyses of the results of efforts to implement ethics guidance for HIV prevention trials.

Here again, there has been a general lack of consolidation and synthesis and a tendency toward replicating the same basic research with each prevention modality and even each trial. Since many of the ethical challenges confronting HIV prevention trials grow out of the social conditions within which the epidemic is embedded, a synthesis of applied ethics experience and findings from the trials could do much to inform the ethical challenges that will confront the development and scale up of combination prevention approaches.

### Social science research opportunities

For the past 20 years, it was arguably premature to devote limited resources to hypothetical social challenges specific to the roll out of yet-unproven interventions, such as vaccines, microbicides and PrEP. That said, many of the hypothetical challenges were in fact already in play, beginning with efforts to roll out condoms (male and female) and to facilitate access to sterile injecting equipment for drug users. We may have lost important opportunities to efficiently explore means for addressing challenges that cross-cut multiple HIV prevention modalities by viewing clinical prevention trials as outside the realm of public health practice.

Biomedical prevention research is now in a transition from a preponderance of trials with flat or negative outcomes to a trend toward trials demonstrating partial effectiveness for specific populations and modes of transmission. With the transition, there is now clear benefit to enhancing the success of biomedical HIV prevention research through development of a coherent programme of social science research that can address known and likely challenges to programmatic success.

### Achieving moral outcomes

An important emerging challenge related to partial effectiveness centres on determining ethical standards of prevention care for future HIV prevention trial participants [44]. Since the early 1990s, appropriate prevention standards have been debated in terms of the potential for exploitation of participant vulnerability and risk, practical implications for efficient clinical trial design, sensitivity to local context (including regulations about provision of injecting equipment for drug users), whether access to otherwise unavailable prevention services could constitute undue inducement for trial participation, and sustainability of the services in the community after the trial. A consensus minimal standard emerged that included client-centred HIV risk reduction counselling, screening for and treatment of curable sexually transmitted infections, and provision of male and female condoms.

Updated ethical guidance from UNAIDS and WHO in 2007 cited a higher standard that includes “all state-of-the-art risk reduction methods”, yet then qualifies this requirement by stating that decisions about the HIV prevention package to be provided should be balanced with the need to ensure that a trial is sufficiently powered to generate an unambiguous result [22].

Inclusion of an increasing number of partially effective interventions in the standard prevention package will likely decrease HIV incidence and, hence, statistical power for evaluating new or potentially more effective interventions. Withholding proven interventions, even if they are not otherwise available to the local population, raises familiar issues of exploitation. This dilemma bears many similarities with the challenges faced in deciding upon an appropriate and feasible standard for ancillary care in biomedical HIV prevention trials, which, as previously noted, benefitted from empirical research with a wide range of stakeholders.

Research would be useful on the long-term effects of how we resolve these issues with regard to individual and group support for, versus opposition to, taking part in trials. Further, informed and comprehensive social analysis of the dilemmas inherent to HIV prevention research would potentially move public dialogue about a wide range of health inequities forward toward global solutions and contribute to the framing of a global public health ethics.

### Understanding, measuring and describing complex human behaviour

“While it is difficult to predict anything with certainty, the evidence suggests that the effectiveness of some (perhaps most) biomedical interventions will vary by gender. Circumcision is the obvious example, but there is the possibility that antiretrovirals for prevention will show complex interactions with biology [45]. Gel-based formulations may be differentially absorbed in the vagina versus the anus. Oral pills may show similar differentiation with regard to drug availability at the locus of virus entry, or they may interact with hormonal contraception. Gender norms may influence efficacy if men and women experience differential rates of blood exposure through the sharing of injection equipment or via iatrogenic means. And, of course, there may be social factors that lead to gender differences in acceptability, adherence and access to interventions that do not vary biologically.

Based on the challenges confronted in providing effective contraceptives to women in resource-poor settings, this has serious implications for the successful roll out of effective HIV prevention for women [46]. Social science research related to gender equity, health policy and implementation practices can inform the development of solutions to the challenges that are likely to emerge. In this regard, important lessons can be learned from a review of the social aspects of efforts to scale up HIV treatment [47].

As placebo-controlled trials become increasingly untenable, the classic randomized controlled trial (RCT) with longitudinal cohorts and HIV infection as the outcome will be severely challenged with regard to statistical power [5]. Instead of a trial with 5000 to 10,000 participants costing $US30 million to $US100 million, a non-placebo controlled trial would likely require tens of thousands of participants and well over $US100 million. By way of example, the RV 144 Phase III HIV vaccine trial enrolled about 16,000 participants in Thailand, lasted about six years, and cost about $US105 million, while the MDP 301 Phase III microbicide trial enrolled 9385 women at multiple sites in Africa and cost $US64 million [48]. The inclusion of multiple partially effective biomedical and behavioural intervention components also means that trial designs are becoming more complex, which further reduces the power of the classic RCT design based on cohorts and incident HIV infections.

Individual behaviour and social context are increasingly recognized as important parameters for biomedical prevention effectiveness [49], [50], [51]. Just as we need to understand the complexities of HIV at the viral level -the genetics, the interaction with human biology at the cellular and systemic levels, the molecular susceptibilities - we need to understand the complexities of HIV at the behavioural and societal levels.

The development and testing of combination prevention and multidisciplinary prevention packages stands to benefit from increased social science leadership. For example, the combination of biomedical interventions, such as tenofovir gel and male circumcision, with social interventions to address gender dynamics and structural interventions to address economic drivers offer the potential to intervene on the epidemic from multiple levels. The design and evaluation of such comprehensive packages will not be easy. But one thing we can learn from the experience of biomedical HIV prevention is the value of perseverance.

### Refraining the social

HIV epitomizes the notion of disease as social ecology, the absolute necessity of understanding context in order to effectively prevent on-going disease transmission. As a sexually transmitted disease, HIV is tightly embedded with not only individual behaviour, but also with behaviours that are at the root of the structuring of human society, including gender, marriage, family, household, kinship, economics and religion. HIV infection is complexly related to HIV exposure; similarly, HIV infectiousness is complexly determined by the interaction of the virus with the human immune system. HIV transmission is not simply an event but a dynamic, evolving process that sends its roots into our complex human systems over the course of years. As a chronic treated condition, those years become decades. HIV is now rooted in the social ecology of thousands of communities and it has demonstrated its capacity to propagate through human generations.

The challenge in reframing the social as a researchable and verifiable component of HIV biomedical prevention is to avoid the trap of oversimplification. The predominant biomedical model for identifying effective HIV prevention strategies persists in modelling transmission as an individual-level event and is built on assumptions of simple linear causality and the independence of effects. Rather than stripping social analysis of its complexity in order to force it into an increasingly limited RCT model, we need to bring the social science tools developed for studying relationships, interdependence and dynamic complexity together with the biomedical tools for studying prevention effectiveness. The end result needs to be a new way of thinking and of doing research, one that integrates social science as a legitimate way of framing problems, designing research to address them, interpreting findings and translating research into action.

## Conclusions

As social scientists, we need to move beyond a critical framing of the social in opposition to the biomedical. We need to ask how the dynamics of social change can be combined with our increasing understanding of the biomedical dynamics of HIV transmission to create, evaluate and implement highly effective HIV interventions. We need the opportunity to work with the complexities of social systems with the same degree of nuance and rigour that biomedical researchers work with the complexities of genetic systems, immune systems and population-level transmission dynamics. Our understanding of each of these systems includes attention to the idiosyncratic as well as the generalizable, the random along with the patterned, the mutable together with the stable. All have much to offer in this endeavour, and much to learn.

## Competing interests

There are no competing interests to declare.

## Author’s contributions

KM contributed to the conception, design, interpretation and drafting of this manuscript and is sole author.
